# Examining Myeloperoxidase (MPO) biomarker in the saliva of patients with *Lactobacillus*-associated caries in Hilla City

**DOI:** 10.25122/jml-2021-0138

**Published:** 2023-07

**Authors:** Hanan Selman Hessan, Baha Hamdi Hakim Al-Amiedi, Reyam Abdul Khuder Mohammed

**Affiliations:** 1 Department of Microbiology, College of Dentistry, University of Babylon, Babylon, Iraq; 2 Medical Laboratory Techniques Department, Al-Mustaqbal University College, Hilla, Iraq

**Keywords:** Myeloperoxidase, caries, *Lactobacillus*, saliva

## Abstract

This study aimed to evaluate the levels of myeloperoxidase (MPO) biomarkers in the saliva of individuals with *Lactobacillus* spp.-related caries in Hilla City. A total of 115 samples were collected, including 90 (78.26%) from patients with severe dental caries and 25 (21.74%) from healthy subjects as a control group. The age range was between 20 and 45 years, with a mean age of 33.76±2.01 for patients with severe dental caries and 24.15±0.44 for healthy subjects. Female participants constituted the majority of the study sample, with 77 (85.5%) females and 13 (14.5%) males. Pathogenic bacteria were isolated and identified using gram staining, biochemical tests, and the VITEK 2 compact system. Of the 90 clinical samples, 65 (72.2%) showed positive bacterial culture, while 25 (27.7%) samples had no bacterial growth. *Lactobacillus* spp. accounted for 25/65 (38.4%) of the total isolates, making it the predominant etiological agent compared to other types of bacteria, constituting 40/65 (61.5%). There was a significant decrease (p≤0.05) in myeloperoxidase levels in patients with severe dental caries and positive *Lactobacillus* culture compared to the control group.

## INTRODUCTION

Myeloperoxidase (MPO) is an antimicrobial enzyme that acts against infectious microorganisms, such as those found in saliva [1]. It is produced and released by human salivary glands and detected in polymorphonuclear (PMN) leukocytes in gingival crevices [2]. The quantity of MPO-containing leukocytes fluctuates in response to changes in salivary gland function, with high levels of HS-LP often indicating infection or inflammation in oral tissues [3]. Saliva samples can be separated into their pure components, including soluble and sedimentary components, and from detergent extracts of the saliva sediment [4]. Lactoperoxidase (L-P), a bovine milk protein, and MPO, a human leukocyte protein, share similar activity characteristics [5].

The aerobic enzyme MPO is present in phagocytes, including neutrophils, monocytes, and macrophages, which play a role in antimicrobial and inflammatory responses [6]. MPO, a hematin enzyme, oxidizes chloride ions by utilizing the oxidizing potential of superoxide and hydrogen peroxide (H2O2) to generate hypochlorous acid and other reactive oxygen species (ROS) [7]. The oral cavity is colonized by a diverse indigenous microflora comprising over 500 species, with the majority remaining uncultivable [8]. Factors contributing to tooth decay include bacterial plaque, diet, and various host factors, such as social, genetic, chemical, and medical conditions [9]. Dental caries is among the most prevalent chronic infectious diseases worldwide [10].

Demineralization and remineralization of dental tissues mark the progression of dental caries, a biofilm-related, sugar-driven, multifactorial, dynamic disease. Caries processes follow a similar pattern, regardless of the type [11]. Streptococcus mutans and lactobacilli species are common in biofilm as they produce weak organic acids during carbohydrate metabolism, which results in tooth demineralization. Cavitation may occur in more than 80 species without impeding the diffusion of calcium, phosphate, and carbonate [12]. Lactobacilli can be classified into two subcategories: homofermenters and heterofermenters. Homofermenters primarily produce lactic acid (65%) from glucose fermentation, such as *L. casei* and *L. acidophilus*, while heterofermenters generate lactic acid, acetate, ethanol, and carbon dioxide by fermenting sugars like glucose [13].

Lactobacilli play a crucial role in forming dental caries lesions, with some suggesting a possible opportunistic function in caries development. Although they can thrive in the acidic environment of deep caries lesions, their presence is associated with the progression of tooth decay, particularly in dentin [14]. This study aimed to evaluate the level of myeloperoxidase (MPO) biomarker in the saliva of individuals with *Lactobacillus* spp.-related caries in Hilla City.

## MATERIAL AND METHODS

### Study design and participants

This cross-sectional study was conducted between July and September 2020 at a private dental clinic in AL-Hilla. A total of 115 participants were enrolled in the study, including 90 patients with severe dental caries and 25 healthy controls. The inclusion criteria for patients with severe dental caries were based on the DMFT (Decayed, Missing, and Filled Teeth) index of the World Health Organization [15]. Both male and female participants aged 20-45 years were included in the study.

### Sample collection and processing

Saliva samples were collected from each participant using disposable cotton swabs following standard procedures for microscopic examination and isolation of bacteria to avoid contamination. To ensure the samples were debris-free, all study participants rinsed their mouths with distilled water (10 mL) for 30-60 seconds. Clean, non-stimulated saliva was collected, placed in sterile laboratory cups, and kept cool using ice packs. The cups were centrifuged at 3000 rpm for 10 minutes to separate unwanted free salivary particles. The clear salivary solution was aspirated using (1 µL) micropipette tips and saved in a 1 mL sterile Eppendorf tube. The samples were frozen at -20°C and used for the final immunological examination study using enzyme-linked immunosorbent assay (ELISA) tests. The instructions from a relevant reference book were followed precisely to collect saliva samples in a contamination-free manner for each patient [16].

### Bacterial identification

The specimens were transferred to the Department of Microbiology for further investigations. Saliva samples were inoculated onto blood agar and selective medium De Man Rogosa Sharpe (MRS) agar and then incubated at 37°C for 24 hours under anaerobic conditions. Anaerobic bacterial isolates were identified using gram stain, colony morphology, biochemical tests, and Compact VITEK-2 System.

### Myeloperoxidase (MPO) activity assay kit

The myeloperoxidase activity assay kit was used to calculate MPO indirectly by measuring the OD value at 460nm. This was achieved by reducing hydrogen peroxide to a complex, which then reacted with o-dianisidine (as a hydrogen donor) to produce a yellow product that had a maximum absorption peak at 460 nm.

### Identification of bacterial isolates by gram stain and biochemical tests

The identification tests, including cultural, morphological, and biochemical characteristics, were done for each isolate according to other relevant studies [17, 18].

### Identification of *Lactobacillus* isolates with Compact VITEK-2 System

Compact VITEK-2 was used to identify and screen all bacterial isolates (BioMerieux). This system is a biochemical identification type that has a VITEK-2 card with 64 wells, each containing a separate fluorescent biochemical test. Twenty tests were used to ascertain carbohydrate assimilation, including phosphatase, urea, nitrate, and actidione assays. The VITEK 2 machine was operated under full control of the incubator, which was maintained at a constant temperature of 35°C, with automatic card filling, sealing, and transfer functions. The obtained results were analyzed using algorithms with a unique coding scheme. The ID-GP (identification of Gram-positive bacteria) databank was used to identify the bacterial isolates. An ID is generated based on the software connected to these systems. In cases where the first test showed “low discrimination” or “no ID,” the test was repeated for analysis, and the findings were used for data.

The cells were incubated overnight at 37°C, and the various strains were injected into the medium. An isolated colony was utilized to make a proper identification using the VITEK-2 Systems technique (BioMerieux). The antibiotic suspension was prepared using the manufacturer's recommendations from BioMérieux, a French pharmaceutical company, by inoculating a sufficient number of overnight pure culture colonies with a sterile swab and transferring the microorganism to a (12x75) mm clear plastic (polystyrene) test tube containing 3.0 ml of sterile saline. Densi Chek was used to measure the turbidity of the liquid and modify it to resemble a McFarland number (0.5). The same suspension was used in the antibiogram tests of the VITEK-2 compact system.

### Data analysis

Descriptive statistics were used to summarize the demographic characteristics of the study participants, including age and gender distribution. The data collected from the saliva samples were analyzed using appropriate statistical methods to assess the levels of myeloperoxidase (MPO) biomarker and the prevalence of *Lactobacillus* spp. in patients with severe dental caries. All statistical analyses were performed using Statistical Package for the Social Sciences (SPSS) version 23. Statistical significance was set at p≤0.05.

## RESULTS

**Figure 1:**
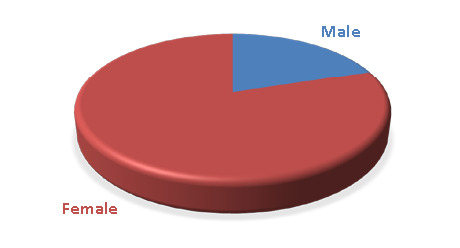
Distribution of patients according to gender

This study collected 115 saliva samples from 90 patients (78.26%) with severe dental caries and 25 healthy (21.74%) controls. The age range of the participants was between 20-45 years, with a mean age of 33.76±2.01 for patients and 24.15±0.44 for healthy controls, as presented in Table 1. The majority of the samples collected in this study were from females, accounting for 77 (85.5%) of the total, while 13 (14.5%) were males, as shown in Table 2 and Figure 1.

**Table 1:** Mean and standard deviation of patients and control groups according to age

Characteristic	Number (%)	Age Mean ± S.D	p-value
**Patients**	90 (78.26)	33.76±2.01	0.0001*
**Control**	25 (21.74)	24.15±0.44	
**Total**	115 (100%)		

* significant difference ≤0.005

**Table 2:** Distribution of patients according to gender

Gender	Number	Percent	x^2^	p
Male	13	14.5%	10.138	≤ 0.05*
Female	77	85.5%		
Total	90	100%		

* significant difference ≤0.005

**Figure 2:**
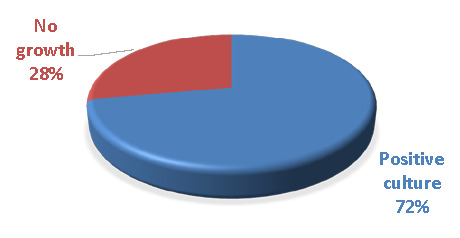
Positive and negative culture of patients with severe dental caries

Out of the 90 collected samples, all were inoculated to identify isolated pathogenic bacteria. Using gram stain, biochemical tests, and the compact VITEK 2 system, 65 samples (72.2%) displayed positive culture, indicating the presence of pathogenic bacteria (Figure 2). However, 25 samples (27.5%) did not show bacterial growth. This may be due to the presence of other types of bacteria that may require special methods for their recognition, such as viruses and fungi, or due to treatment with antibiotics.

**Figure 3:**
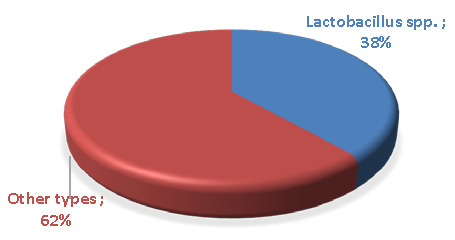
Detection of *Lactobacillus* spp. isolates from patients

Among the isolated bacteria, *Lactobacillus* spp. were the most common, constituting 25/65 (38.4%) of the total isolates, and were considered the predominant etiological agent compared to other types of bacteria, constituting 40/65 (61.5%). The detection of *Lactobacillus* spp. isolates from patients are shown in Table 3 and Figure 3.

**Table 3:** Detection of *Lactobacillus* spp. isolates from patients with severe dental caries

Total No. of clinical samples	Positive culture	*Lactobacillus* spp.	Other types	No growth
90 samples	65 (72.2%)	25/65 (38.4%)	40/65 (61.5%)	25 (27.7%)

### Myeloperoxidase (MPO) biomarker levels in patients with a positive culture of *Lactobacillus* spp

**Figure 4:**
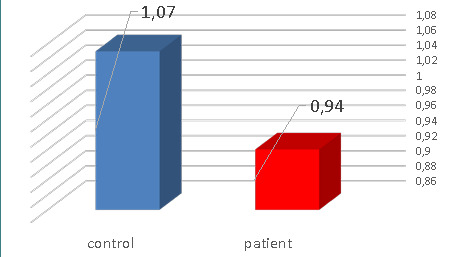
Mean and standard deviation of MPO biomarker level in patients with positive culture for *Lactobacillus* spp. and control groups

We also investigated the level of MPO biomarker in patients with positive culture for *Lactobacillus* spp. compared to the control group. The mean differences in myeloperoxidase biomarkers between study groups are presented in Table 4 and Figure 4. The results showed a significant decrease (0.94±0.34) (p≤0.05) in MPO in patients with severe dental caries and positive culture for *Lactobacillus* compared to the control group (1.07±0.34) (p≤0.05).

**Table 4:** Mean and standard deviation of MPO level in patients with positive culture for *Lactobacillus* spp. and control groups

Biomarker	Patients group No=25	Control group No.=25	p-value
	Mean ± S.D	Mean ± S.D	
MPO	0.94±0.34	1.07±0.34	0.0001*

* significant difference ≤0.005

## DISCUSSION

Research has shown that caries risk factors differ between men and women, which may explain the gender disparity in caries rates. Unique factors such as saliva composition and flow rate, hormonal fluctuations, dietary habits, genetic variations, and specific social roles within the family can all contribute to higher caries rates in females compared to males, as reported by Wang *et al*. [19]. Additionally, Lira-Junior *et al*. [20] found a higher prevalence of caries-related diseases in females, which may also contribute to their higher caries rates [20]. Milona *et al*. [21] also observed a higher proportion of female patients compared to males, with significant differences.

This study found that individuals aged 25 to 45 had a higher incidence of caries formation. This finding is consistent with previous research by Ortiz *et al*. [22], who reported an increase in plaque formation during childhood and teenage years. The findings of this study align with previous research by Rowińska *et al*. [23], which suggests that caries development is a gradual process and that consuming sugary foods, such as sweets and sticky snacks, is relatively high among teenagers. Additionally, Tonetti *et al*. [24] noted that dental caries is a cumulative process that worsens with age, and untreated caries in high-risk individuals are likely to progress more rapidly than in younger individuals, often leading to tooth extraction. Another possible explanation for the higher incidence of caries among older individuals is the increased likelihood of experiencing dental pain. Patients in this age group often have fixed dentistry appliances for both esthetic and practical reasons, which can contribute to excessive caries, as reported by Hasan *et al*. [25]. The current study showed that the fixed treatment period had a powerful result on plaque formation.

Kearney *et al*. [26] found a high caries prevalence and high DMFT index among adults who skip breakfast and are likelier to snack throughout the day. Snacks typically have the highest sugar content of any meal type (e.g., breakfast, lunch, dinner). Factors such as sex, subjective socioeconomic status (SES), regular dental visits, and skipping tooth brushing were identified as essential determinants of dental caries [9]. Blostein *et al*. [27] reported similar findings. Tan *et al*. [28] reported a prevalence rate of 70.8% for *Eubacterium*-related caries cases, while Mbabazi [29] found that 35% of dental caries cases were caused by bacteria, with about 65% being *Lactobacillus* and 35% other species. The ecology of lactobacilli in the oral cavity was studied by Kearney *et al*. [30], who reported that *Lactobacillus* is the primary microorganism involved in dental caries development.

The study by Ahirwar *et al*. [31] also detected an increase in the proportion of lactobacilli before the onset of carious lesions. Lactobacilli played a crucial role in plaque formation, potentially leading to caries development. The higher frequency of *Lactobacillus* infection was due to lower educational attainment, primary cycle students, a sugar-rich diet, poor teeth-cleaning habits, dental discomfort, and the acidity scale for dental caries being a determining factor, as reported by Ademe *et al*. [32]. In their study, Mahasneh and Mahasneh [33] confirmed that Eubacterium is also associated with dental caries development. The occurrence of dental caries depends on the balance of microorganisms in the oral cavity.

The ability of *Lactobacillus* to adhere to tooth enamel is a critical factor in the development of dental caries, as reported by Philip [34]. Khan *et al*. [35] found that MPO deficiency affects approximately 1 in 2000 to 1 in 4000 individuals. Dijkstra *et al*. [36] reported that MPO deficiency could lead to increased inflammation or infection complications. Al-Shehri [37] noted that the antibacterial and candidacidal actions of MPO-deficient neutrophils are suboptimal and inadequate against certain bacteria and fungal species.

MPO deficiency is an inherited disease associated with decreased immunity [38]. Kunnumakkara *et al*. [39] reported that various autoantibodies, including MPO antibodies, are present in inflammatory processes. Myeloperoxidase has also been suggested as a potential inflammation biomarker in various diseases such as anemia, heart disease, and acute coronary syndrome [40]. As inflammation reaches a certain threshold, the permeability of vascular structures increases dramatically, allowing immunoglobulins and fluid proteins to flow to the inflammation site, as stated by Stankova *et al*. [35, 41]. MPO release is triggered by the cascade of inflammatory processes, as noted by Pizzolo *et al*. [29, 42].

## CONCLUSION

The findings of this study suggest that females may be at a higher risk for dental caries, as they were the most commonly affected patients in the dental clinic. *Lactobacillus* was found in a significant proportion of caries cases, with a rate of 38.4%. Additionally, the concentration of MPO biomarkers in the saliva of tooth decay patients was found to be significantly lower compared to the control group, indicating a potential role of MPO in caries prevention.
